# A Novel DC Therapy with Manipulation of MKK6 Gene on Nickel Allergy in Mice

**DOI:** 10.1371/journal.pone.0019017

**Published:** 2011-04-22

**Authors:** Megumi Watanabe, Naozumi Ishimaru, Meinar Nur Ashrin, Rieko Arakaki, Akiko Yamada, Tetsuo Ichikawa, Yoshio Hayashi

**Affiliations:** 1 Department of Oral Molecular Pathology, Institute of Health Biosciences, The University of Tokushima Graduate School, Tokushima, Japan; 2 Department of Oral Maxillofacial Prosthodontics, Institute of Health Biosciences, The University of Tokushima Graduate School, Tokushima, Japan; Centre de Recherche Public de la Santé (CRP-Santé), Luxembourg

## Abstract

**Background:**

Although the activation of dermal dendritic cells (DCs) or Langerhans cells (LCs) via p38 mitogen-activated protein kinase (MAPK) plays a crucial role in the pathogenesis of metal allergy, the *in vivo* molecular mechanisms have not been identified and a possible therapeutic strategy using the control of dermal DCs or LCs has not been established. In this study, we focused on dermal DCs to define the *in vivo* mechanisms of metal allergy pathogenesis in a mouse nickel (Ni) allergy model. The effects of DC therapy on Ni allergic responses were also investigated.

**Methods and Finding:**

The activation of dermal DCs via p38 MAPK triggered a T cell-mediated allergic immune response in this model. In the MAPK signaling cascade in DCs, Ni potently phosphorylated MAP kinase kinase 6 (MKK6) following increased DC activation. Ni-stimulated DCs could prime T cell activation to induce Ni allergy. Interestingly, when MKK6 gene-transfected DCs were transferred into the model mice, a more pronounced allergic reaction was observed. In addition, injection of short interfering (si) RNA targeting the MKK6 gene protected against a hypersensitivity reaction after Ni immunization. The cooperative action between T cell activation and MKK6-mediated DC activation by Ni played an important role in the development of Ni allergy.

**Conclusions:**

DC activation by Ni played an important role in the development of Ni allergy. Manipulating the MKK6 gene in DCs may be a good therapeutic strategy for dermal Ni allergy.

## Introduction

Metal allergy is an inflammatory disease categorized as a delayed-type hypersensitivity (DTH) reaction, similar to contact dermatitis and eczema [1], [2]. This skin disease is induced by a complex process involving immune responses of numerous cell types, and cooperation among these cells is crucial for symptom development [3], [4].

Among various metals, nickel (Ni), when used in costume jewelry or dental alloys, is the most frequent cause of contact allergy [5]–[7]. Ni-specific T cell responses are crucial for the development of allergies in human and mouse models [8]–[10]. However, it is unclear how T cells recognize Ni presented to them by antigen-presenting cells (APCs). In the skin, Langerhans cells (LCs) or dermal dendritic cells (DCs) play fundamental roles as APCs for uptake, processing, and presentation of antigens [11], [12]. Although there is no evidence that DCs or LCs can directly clear Ni, these APCs may contribute to a Ni allergic response via other molecular mechanisms.

Ni ions (Ni^2+^) are known to be released from various alloys into the skin and exert proinflammatory and irritant properties as potent allergens or haptens [13], [14]. Ni penetrates the skin where it may associate with epithelial cells or become attached to MHC molecules on LCs or DCs. APCs are activated by certain cytokines such as IL-1β and TNF-α produced by keratinocytes. The cytokines regulate the expression of E-cadherin and chemokines like matrix metalloproteinase (MMP)-9, secondary lymphoid tissue chemokine (SLC), and MIP-3β produced by the APCs [15]–[18]. Subsequently, APCs migrate to draining lymph nodes where they present haptens to naïve T cells. Re-exposure to the same hapten leads to a hypersensitivity reaction in an effector phase.

Recent *in vitro* experiments have reported that contact sensitizers like Ni activate epidermal DCs or LCs as shown by the upregulation of CD80, CD83, CD86, and MHC class II [19]. Moreover, *in vitro* experiments using human DCs showed that Ni-induced phosphorylation of p38 mitogen-activated protein kinase (MAPK) was critical for the maturation of immature DCs [20]–[22]. In addition, the conditional induction of a dominant active form of MAP kinase kinase 6 (MKK6) efficiently induced the activation of human LCs [23]. However, it remains uncertain whether the *in vivo* activation of DCs in the skin is induced by Ni via the MAPK signaling pathway. Furthermore, it is unclear whether the signaling pathway of DCs stimulated by Ni is different from that of the other stimuli with regard to signal strength or the pathway itself.

The aim of this study was to determine the signaling pathway for APC activation in dermal immune responses related to the pathogenesis of Ni allergy in a mouse model. In addition, a therapeutic strategy based on the *in vivo* molecular mechanisms of Ni allergy was applied to this model.

## Results

### Induction of Hypersensitive Reactions to NiCl_2_

**Figure 1:**
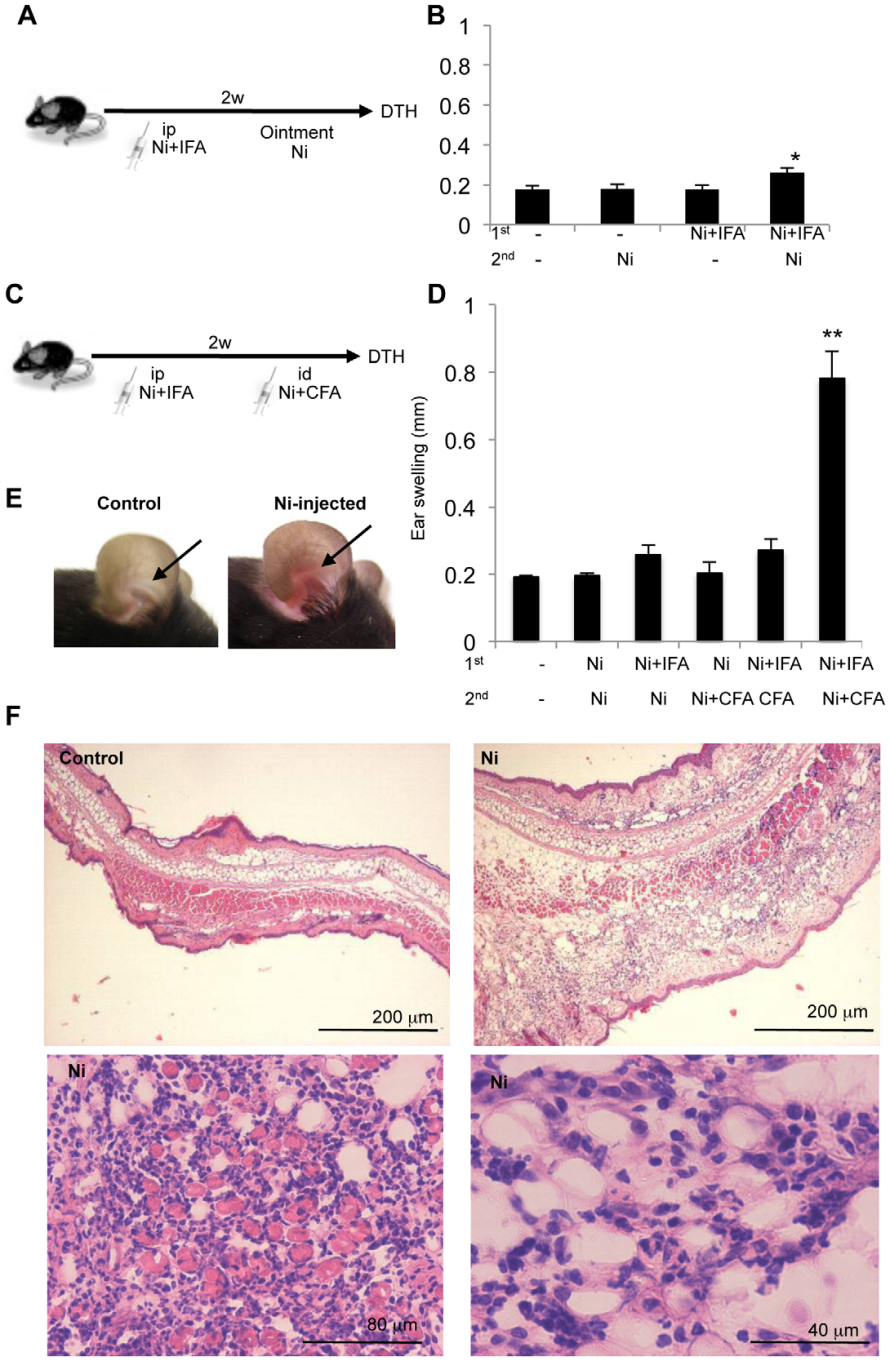
Induction of Ni allergy. (A) Protocol for inducing the Ni allergy model. First, an intraperitoneal injection of NiCl_2_ with or without IFA was administered. After 2 weeks, an ointment (o) of NiCl_2_ with Vaseline was applied to the ear pinnae as a secondary challenge. (B) DTH was assessed by measuring the ear thickness at 48 hours after the last challenge. Results are means ± SD for 4 mice in each group. (C) Protocol for inducing a Ni allergy model. First, an intraperitoneal injection of NiCl_2_ with or without IFA was administered. Then, after 2 weeks, an intradermal injection of NiCl_2_ with or without CFA was administered to the ear pinnae to induce a secondary reaction. (D) DTH was assessed by measuring the ear thickness at 48 hours after the last challenge. Results are means ± SD for 4 to 6 mice in each group. **P<0.005. (E) Representative photos of the ears from control and NiCl_2_-injected mice. (F) Histological images of inflammatory lesions in the skin are representative of 5 mice in each group.

To induce hypersensitive reactions to Ni, Ni is typically applied to the skin surface as a secondary challenge after sensitization. In the first series of experiments, we evaluated results of the mouse ear swelling test, as described previously [24]. Using this protocol (Figure 1A), we found flare reactions and slight increases in ear swelling in response to NiCl_2_ (Figure 1B).

In order to elucidate the symptoms associated with hypersensitive reactions to NiCl_2_, we administered intradermal injections into the ear pinnae of mice as previously described [25]. Although attempts to induce Ni allergy in mice have often failed, a DTH reaction to Ni has been achieved after injecting NiCl_2_ in combination with an adjuvant or irritant [25]. Thus, we attempted to induce DTH by injecting NiCl_2_ in combination with either incomplete Freund's adjuvant (IFA) or complete Freund's adjuvant (CFA) (Figure 1C). A DTH reaction to NiCl_2_ was induced in C57BL/6 (B6) female mice using the method shown in Figure 1C.

Briefly, NiCl_2_ with IFA was intraperitoneally injected into B6 mice for initial immunization. Two weeks later, NiCl_2_ together with CFA was intradermally injected into the ear skin for a recall immune response. DTH reactions were determined by measuring the changes in ear thickness 48 hours after the challenge.

Although increased ear thickness of Ni-treated mice after injection of Ni with IFA has been previously reported [25], in this study, ear swelling was only around 0.2 to 0.3 mm (Figure 1D). In contrast, the ear thickness of Ni+CFA-treated mice after Ni with IFA was significantly higher compared with that of CFA-treated and other mice (Figure 1D). Redness and swelling of the ear skin was observed in Ni+CFA-treated mice after Ni with IFA (Figure 1E). Histological examination of the ear epidermis of Ni+CFA-treated mice after Ni with IFA showed edema, congestion, and extensive infiltration of inflammatory cells, including mononuclear cells, monocytes, neutrophils, and macrophages, in the connective and muscular tissues; however, there was no inflammation in control ears (Figure 1F, Figure S1A). In addition, toluidine blue-positive cells, including degranulated mast cells, found in the lesions of Ni allergy mice were significantly increased compared with those in control mice (Figure S1B). This Ni allergy model with severe inflammation was used to analyze the cellular mechanisms and to develop a therapeutic strategy.

### Cellular Mechanisms of Ni Allergy

**Figure 2:**
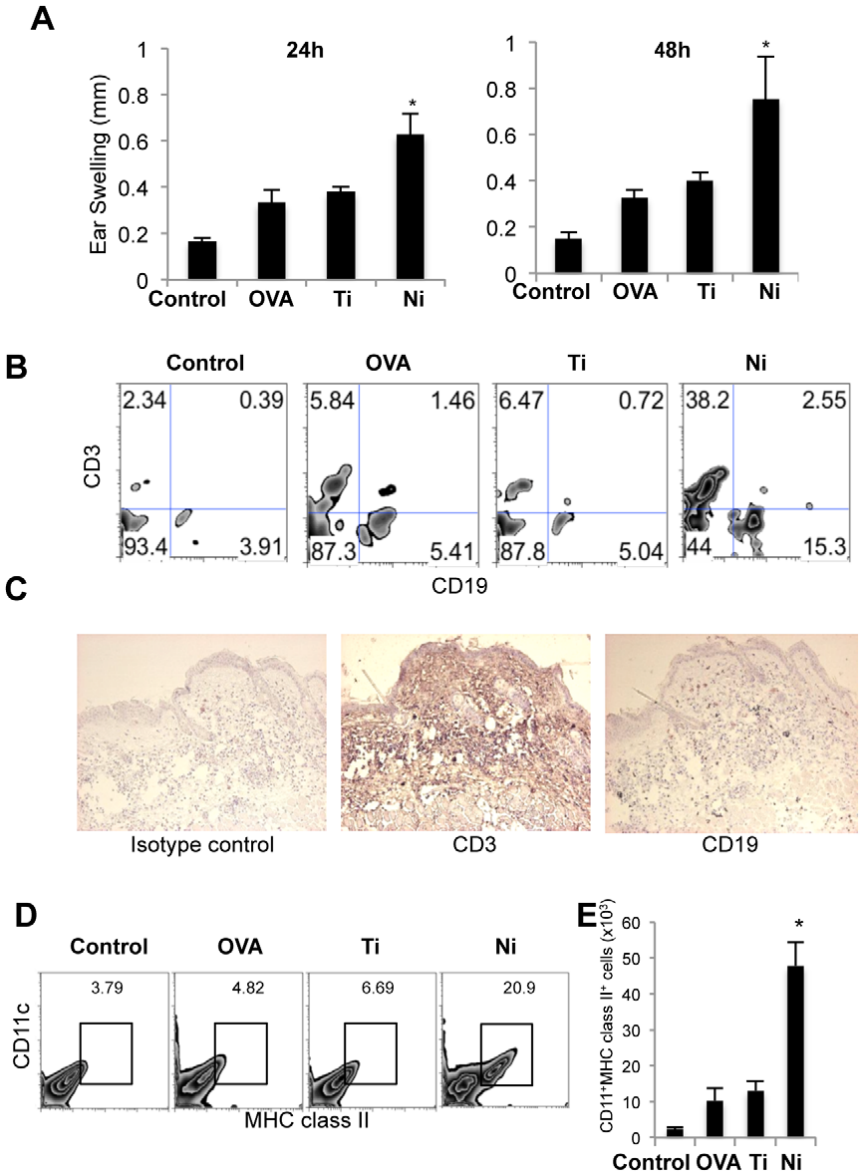
Characteristics of the Ni allergy model. (A) DTH was evaluated by measuring the ear thickness of the treated mice. Results are means ± SD for 5 mice in each group. *P<0.05. (B) T and B cell populations of purified lymphocytes from skin tissues were analyzed by flow cytometry. Representative results of 5 mice in each group are shown. (C) Immunohistochemical analysis of T and B cells was performed on frozen sections of inflamed ear tissues obtained from Ni allergy mice. Representative photos of 2 independent experiments are shown. (D) Dermal DCs were detected by flow cytometry. These results represent 2 independent experiments with 3 mice in each group. (E) The absolute numbers of dermal DCs were determined. *P<0.05.

To characterize Ni allergy, immune responses to another antigen or metal were compared with Ni immune responses in the present allergy model. Briefly, NiCl_2_ or the control metal/antigen (PBS, ovalbumin [OVA], or TiO_2_ [Ti]), along with IFA, was intraperitoneally injected into B6 mice. Titanium has been known as one of biocompatible metals, and so it is believed that the allergic sensitivity induced by nickel is rarely occurred by titanium [26]. Therefore, titanium was used as a control metal in this study. Two weeks later, NiCl_2_ or the abovementioned control metal/antigen, along with CFA, was intradermally injected into the mice. At 24 and 48 hours after immunization, DTH reactions were assessed by measuring ear thicknesses. In contrast to the swelling seen in OVA- or Ti-treated mice, the ear thicknesses of Ni-treated mice were significantly increased at both 24 and 48 hours after the second challenge (Figure 2A).

Flow cytometry was performed to identify the phenotypes of infiltrating lymphocytes in the allergic lesions. Large numbers of CD3^+^ T cells and CD19^+^ B cells were detected in the tissue samples obtained from NiCl_2_-injected mice (Figure 2B). In addition, immunohistochemical analysis indicated that a much higher proportion of CD3^+^ T cells were observed in the skin tissues of NiCl_2_-injected mice, compared with CD19^+^B cells (Figure 2C). The infiltrating T cells were largely CD4^+^ T cells, with small numbers of CD8^+^ T cells and NK1.1^+^ natural killer (NK) cells (Figure S2A). Moreover, the proportion of NKT cells reactive to anti-α-GalCer-CD1d complex in the ear tissues of NiCl_2_-injected mice was significantly increased compared with that of control mice (Figure S2B). The number of MHC class II^+^CD11c^+^ dermal DCs was significantly higher in NiCl_2_-injected mice than in control mice (Figure 2D). In addition, the absolute numbers of dermal DCs in NiCl_2_-injected mice were significantly increased compared with those in control mice (Figure 2E). In contrast, the number of DCs in the cervical lymph nodes of Ni-injected mice was not increased (Figure S2C). These results suggest that T cells and dermal DCs in the skin lesions play important roles in the development of the hypersensitivity reaction induced by NiCl_2_.

### Activation of DCs by NiCl_2_ via MAPK

**Figure 3:**
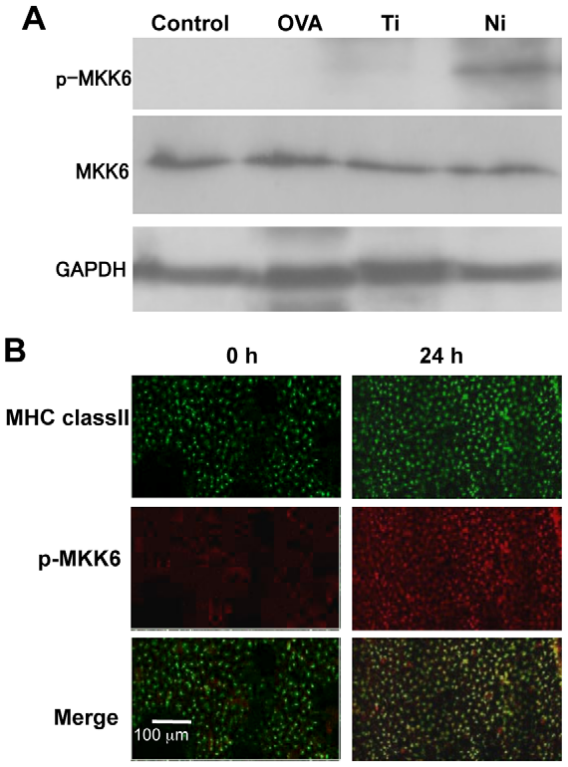
DC activation by Ni via MKK6. (A) Phosphorylation of MKK6 in the inflamed tissues of the Ni allergy model was analyzed by Western blot analysis. GAPDH was used as the internal control. (B) Phosphorylation of MKK6 (Red) of MHC class II^+^ (Green) dermal DCs from Ni allergy mice was detected by confocal microscopy. These results represent 2 independent experiments with 3 to 5 mice in each group.

Numerous signaling molecules control the maintenance of DCs in peripheral tissues [27]. Among these, MAPK is known to be a key signal transducer for the activation of DCs in various immune responses [28], [29]. Regarding DCs in allergic reactions in humans, p38/MKK6 in the MAPK pathway plays a significant role in the activation of DCs during the development of skin allergy [23]. Thus, we focused on p38/MKK6 of DCs in mice. To define *in vivo* p38/MKK6 activation of dermal DCs in our Ni allergy model, Western blot analysis of MKK6 was performed using skin tissue samples. MKK6 phosphorylation was significantly increased in the skin tissues of NiCl_2_-injected mice (Figure 3A), but absent in the skin tissues of PBS-, OVA-, and TiO_2_-injected mice (Figure 3A). Further, phospho-MKK6 was detected in MHC class II^+^ DCs in the skin tissues of NiCl_2_-injected mice (Figure 3B). These results showed that dermal DC activation via MKK6 was important for the pathogenesis of Ni allergy in the skin.

On the other hand, when phosphorylation of MKK6 and p38 in bone marrow-derived cells (BMDCs) stimulated with NiCl_2_ was examined by Western blot analysis, the phosphorylated levels of MKK6 and p38 in NiCl_2_-stimulated BMDCs were elevated similar to those in LPS-stimulated BMDCs (Figure S3A). When stimulated with TiO_2_, however, no phosphorylation of MKK6 and p38 was detected (Figure S3A). We used real-time PCR to examine the MKK6 mRNA expression in BMDCs stimulated with NiCl_2_. The mRNA expression level of MKK6 in BMDCs stimulated with NiCl_2_ was increased in a time-dependent manner; it was much higher than that in LPS-stimulated BMDCs during the first 24 hours (Figure S3B), but decreased after 36 hours (Figure S3B). These results showed that Ni could directly trigger the activation of DCs via p38/MKK6 to induce an allergic immune reaction.

### Enhanced Allergy after Transfer of T Cells Primed by NiCl_2_-stimulated DCs

As shown in Figure 2, a large number of T cells had infiltrated into the lesions of inflamed skin tissues. In addition, when purified T cells from the lymph nodes of the Ni allergy model were stimulated with anti-CD3 mAb, production of Th1-type cytokines, including IL-2 and IFN- γ, was significantly increased compared with that in controls (Figure S4). On the other hand, the secretion of Th2-type cytokines such as IL-4 and IL-10 from anti-CD3 mAb-stimulated T cells of Ni allergy model was similar to that of the other control mice (Figure S4). These findings suggest that T cells play a key role as effector cells in the pathogenesis of Ni allergy. However, it was still unclear how Ni-stimulated DCs were related to T cell responses in allergic reactions.

**Figure 4:**
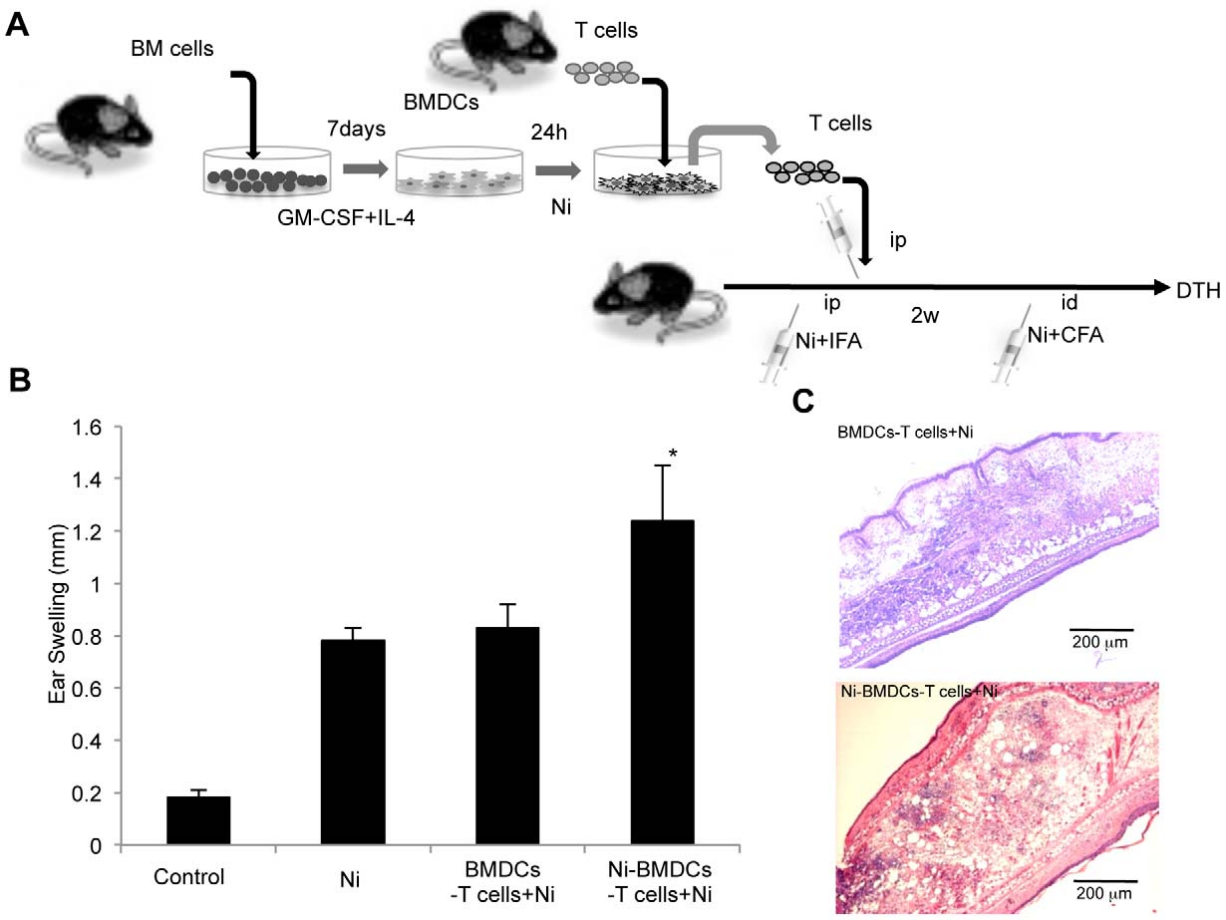
Effect of T cell transfer on DTH in the Ni allergy model. (A) Purified T cells from the spleens of B6 mice were co-cultured with BMDCs in the absence (control) or presence of NiCl_2_ for 48 hours and then intraperitoneally transferred into B6 mice. DTH reaction was induced by injecting NiCl_2_. (B) Ear thickness was measured at 48 hours after the challenge. Results are means ± SD for 5 mice in each group. *P<0.05. (C) Inflammatory lesions of the mice with transferred Ni-DC-stimulated T cells.

Thus, T cells purified from the spleens of normal B6 mice were co-cultured with Ni-stimulated BMDCs *in vitro* for 24 hours. Then, the resulting primed T cells were transferred intraperitoneally into normal B6 mice, and 2 weeks later, NiCl_2_ with CFA was injected intradermally to induce DTH (Figure 4A). The skin thickness of the model mice with transferred Ni-BMDCs-stimulated T cells was significantly increased compared with that of mice with transferred nonstimulated T cells (Figure 4B). Pathological examination of the skin lesions in the mice with transferred Ni-BMDCs-stimulated T cells showed more severe inflammation, including lymphocyte infiltration, edema, and congestion, compared with those in the mice with transferred nonstimulated T cells (Figure 4C). These results suggested that the T cells primed by Ni-stimulated DCs were important for the onset of Ni allergy.

### Effects of Overexpression of MKK6 in DCs on Ni Allergy

**Figure 5:**
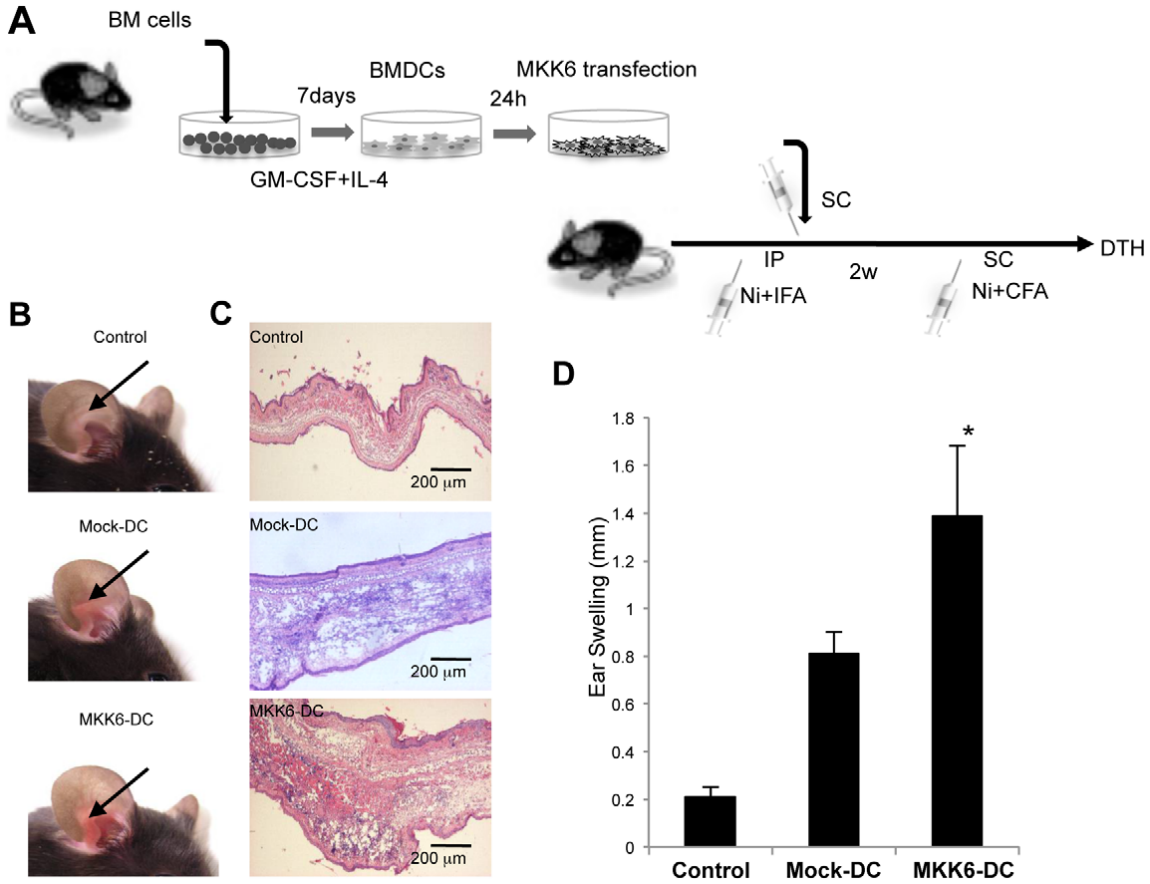
Effect of DC transfer on the Ni allergy reaction. (A) MKK6 DNA or mock plasmids were transfected into BMDCs. MKK6/DCs or Mock/DCs were subcutaneously injected into B6 mice. The mice were challenged with NiCl_2_ to induce DTH. (B) Representative photos are shown for 3 to 5 mice in each group. (C) Inflammatory lesions of the mice with transferred MKK6/DCs. These results are representative of 2 independent experiments with 3 mice in each group. (D) Ear thickness was measured at 48 hours after the challenge. Results are means ± SD for 5 mice in each group. *P<0.05.

To examine whether DCs activated by the engagement of p38/MKK6 signaling influenced the development of skin allergy, BMDCs were transfected with the MKK6 gene (MKK6-DC) and stimulated with NiCl_2_ for 48 hours. Then, MKK6-DCs were subcutaneously transferred into normal B6 mice, and 2 weeks later, NiCl_2_ was intradermally injected to induce Ni allergy (Figure 5A). The skin redness in MKK6-DC-transferred mice was enhanced compared with that in Mock-DC-transferred mice (Figure 5B). In addition, pathological examination revealed remarkable infiltration of immune cells along with congestion and edema in the skin lesions of MKK6-DC-transferred mice (Figure 5C). Significantly enhanced ear swelling was detected within 48 hours of injecting NiCl_2_ into MKK6-DC-transferred mice compared with Mock-DC-transferred mice (Figure 5D). In addition, we investigated production of cytokines by Ni-stimulated DCs that are required for T cell priming, including IL-12, IFN-γ, and IL-10. The amounts of IL-12, IFN-γ, and IL-10 produced by BMDCs after stimulation with NiCl_2_ were equal to those after stimulation with LPS (Figure S5), and the cytokine production by NiCl_2_-stimulated BMDCs were significantly higher compared with that by TiO_2_-stimulated BMDCs (Figure S5). These results suggested that Ni-mediated DC activation played a central role in T cell priming for the onset and development of Ni allergy.

### Effective Therapy for Ni Allergy using MKK6 siRNA

**Figure 6:**
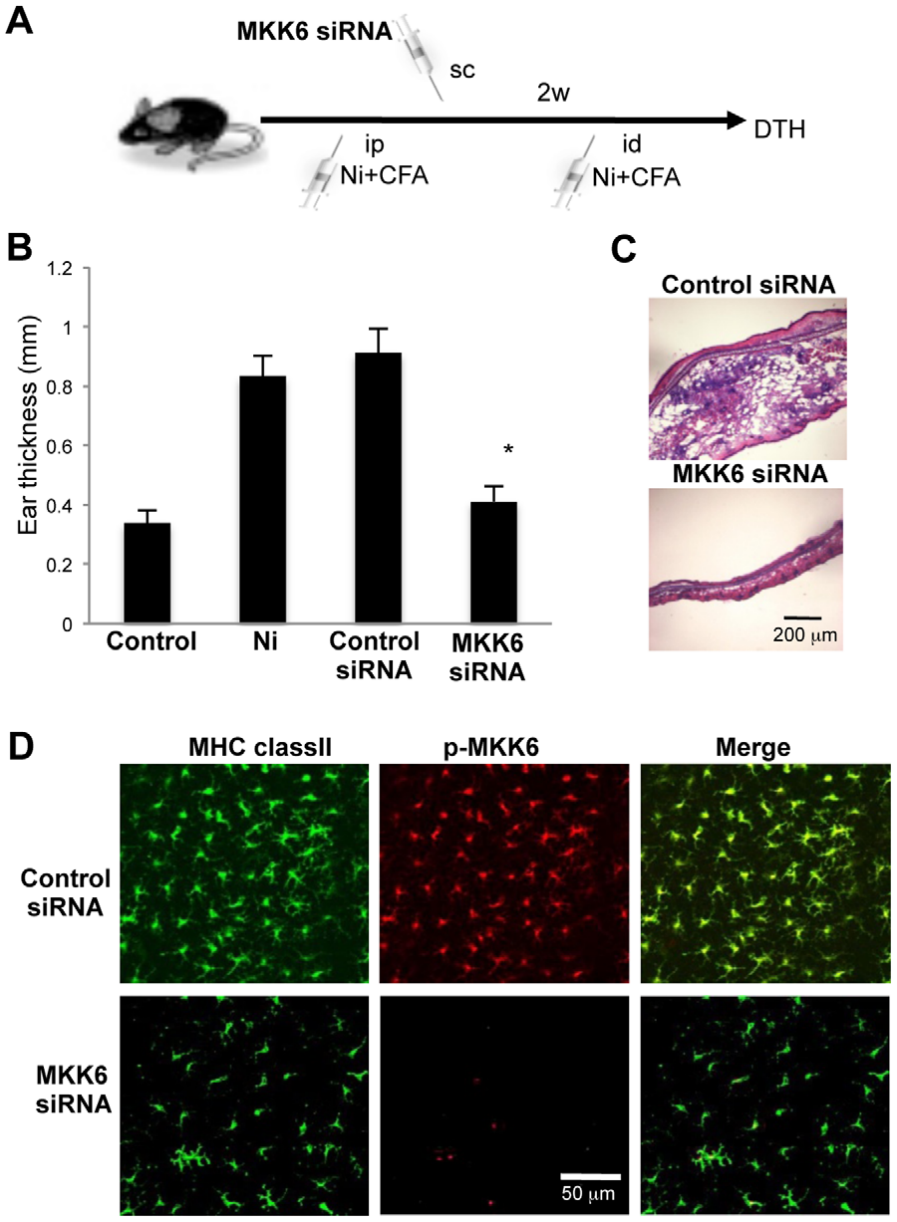
DC therapy for Ni allergy. (A) siRNA for MKK6 with atelocollagen was subcutaneously injected into the ear skin of Ni allergy mice. (B) Ear thickness was measured at 48 hours after injecting NiCl_2_. Results are means ± SD for 5 mice in each group. *P<0.05. (C) Pathological findings after *in vivo* administration of MKK6 siRNA and control siRNA. (D) Phosphorylation of MKK6 of MHC class II^+^ dermal DCs was analyzed by confocal microscopy. These results are representative of experiments with 5 mice in each group.

We next tested a therapeutic strategy for Ni allergy using short interfering (si) RNA targeting the MKK6 gene. Before inducing a DTH reaction, MKK6 siRNA with atelocollagen as an *in vivo* gene delivery system was subcutaneously injected into the ear skin of the model mice. Ear thickness was measured at 48 hours after injecting NiCl_2_ or saline control (Figure 6A). We confirmed that the MKK6 mRNA level in siRNA-treated DCs was significantly reduced (<1/6) compared with that in control siRNA-treated DCs (data not shown).

Ear thickness of MKK6 siRNA-treated allergy model mice was significantly reduced compared with those of control siRNA-treated mice (Figure 6B). In addition, pathological examination showed no detectable inflammatory lesions in MKK6 siRNA-treated mice, while severe inflammation with extensive lymphocytic infiltration, edema, and congestion was observed in the lesions of the mice injected with control siRNA (Figure 6C). Further, although phosphorylation of MKK6 of MHC class II^+^ dermal DCs in the skin sheets from MKK6 siRNA-treated mice was nearly undetectable, there was phosphorylation in the sheets from control siRNA-treated mice (Figure 6D). This showed that an effective therapy using siRNA targeting the MKK6 gene could be used for treating Ni allergy.

## Discussion

DCs in the epidermis and dermis participate in the recognition of pathogens. The two major populations of DCs present in normal skin are epidermal LCs and dermal DCs. These DCs function as APCs and play key roles in sensing pathogens and initiating allergic responses. Immature DCs, such as epidermal LCs, reside in the peripheral tissues. When these DCs are activated by antigens and mature, they migrate from peripheral sites to lymphoid organs where they stimulate T cells to induce an immune response [11], [30]. The cutaneous immune system depends on multiple cell-cell contacts for antigen recognition and presentation, as well as inflammation. LCs facilitate the development of contact hypersensitivity responses by efficiently presenting haptens to T cells [31]. However, it has been suggested that dermal DCs can support LC functions [31].

From our results and those of other studies, allergy symptoms, such as swelling and flare reactions, were minimal and transient after applying a Ni-based ointment to the skin surface. Artik et al. demonstrated contact hypersensitivity in model mice after intradermally injecting Ni into the ear pinnae [25]. Our method using intradermal injections of Ni with CFA in a new DTH model was based on their report. Clinical skin tests (such as puncture tests and intradermal injection tests) along with patch tests have been used for the diagnosis of metal allergy. Therefore, we injected NiCl_2_ with CFA intradermally into mice in order to obtain clear hypersensitivity responses.

p38 MAPK is an evolutionarily highly conserved stress response pathway in various cells [32]. p38 MAPK is activated by an upstream kinase (MKK6 or MKK3) and then translocated into the nucleus where target molecules are phosphorylated by p38 MAPK [33], [34]. A previous report showed that activation of human LCs was triggered by MKK6 [23]. In addition, several reports using *in vitro* experiments showed that p38 MAPK phosphorylation played an important role in the activation of DCs by Ni [34]–[36]. Our experiments using a Ni allergy model strongly suggest that MKK6 phosphorylation in dermal DCs is the first important trigger for the onset of an allergic response to Ni.

Ni is the most frequent cause of metal allergy. It is possible that Ni in the skin may interact with proteins, after which dermal DCs capture the Ni–antigen complex. At this stage, Ni might be a potent factor for triggering activation of DCs via MKK6. In our model, the specific activation of MKK6 in DCs was observed only when the cells were stimulated with Ni but not another metal or antigen such as Ti or OVA. However, because the phosphorylation of MKK6 in DCs was detectable after stimulation with LPS *in vitro*, the signaling pathway induced by Ni could be modulated by other stimuli. In addition, we could not identify the mechanism by which Ni modulated MKK6 expression.

It was recently reported that human Toll-like receptor (TLR) 4 plays a crucial role in the development of contact allergy to Ni [37] and that only TLR4-deficient mice expressing transgenic human TLR4 developed contact hypersensitivity to Ni, while animals expressing mouse TLR4 did not develop DTH. Although the cell type contributing to a TLR4-mediated allergic reaction was not identified, immune cells such as DCs, macrophages, and endothelial cells were associated with an allergic reaction to Ni via TLR4. It is possible that TLRs other than TLR4 are related to Ni allergy in mice. In our mouse model, although the signaling pathway via TLR in DCs was not examined, MKK6 phosphorylation was observed in LPS-stimulated DCs *in vitro*. Thus, cooperative or synergistic actions of Ni with other signaling pathways, including TLR, following MKK6 activation may be associated with Ni allergy in this mouse model. Our *in vivo* experiments suggested that DCs activated by Ni enhanced T cell migration to local lesions and T cell-dependent allergic response; however, it remains unclear how Ni can stimulate or control DC activation via MKK6. Moreover, our result showed that significantly increased number of NKT cells reactive to anti-ααGalCer-CD1d complex was found in the ear lesion of Ni model mice. CD1d-restricted NKT cells have been referred to as natural memory cells in both innate and acquired immune responses [38]. However, the relationship between metal allergy and the role of NTK cell has been still unidentified. Further research using our model will be necessary for understanding the cellular mechanism of metal allergy.

DC maturation can be initiated by inflammatory stimuli, such as cytokines, LPS, CD40 ligation, and contact allergens [39]. Activated DCs take up antigens and produce a variety of cytokines and chemokines, which in turn attract and activate eosinophils, macrophages, and NK cells to the site of antigen entry [30]. After antigen capture, DCs migrate to regional lymph nodes and present peptide-MHC complexes to antigen-specific T cells that induce T cell-dependent immune responses, such as Ni allergy [40]. Interestingly, our results showed a significantly increased number of dermal DCs in the skin tissues of mice with Ni allergy. These findings suggest that circulating precursor DCs might accumulate at the NiCl_2_ injection site. Moreover, the experiments using skin tissues demonstrated that phosphorylation of MKK6 in dermal DCs and LCs was clearly detectable. These results indicated that stimulation with Ni enhanced both accumulation and activation of DCs.

DCs are activated by signaling via pattern recognition receptors (PRRs), such as TLRs and retinoic acid-inducible gene I-like receptors, in response to a variety of ligands [41], [42]. PRR signaling leads to the activation of nuclear factor (NF)-κB. Although it is unclear whether Ni can interact with receptors such as PRRs, previous reports showed that the differentiation of human DCs is promoted by Ni via NF-κB activation [43], [44]. It remains uncertain whether NF-κB signaling plays a role in the pathogenesis of metal allergy.

Our *in vivo* experiments demonstrated that manipulating the MKK6 gene of DCs could control Ni allergy. Many reports on DC therapy for cancer or infection have indicated that antigen presentation to T cells by DCs is important for controlling these diseases [14], [45], [46]. However, there are no reports regarding DC therapy for metal allergy. Our new approach with siRNA targeting the MKK6 gene could be a powerful strategy for the prevention and cure of metal allergy. Careful attention should be paid to the therapeutic effects of MKK6 inhibition on the immune system in the treatment of allergic immune responses.

In conclusion, the signaling pathway via p38/MKK6 plays a key role in activating dermal DCs in the pathogenesis of metal allergy. DC therapy using MKK6 gene manipulation could be effective for treating metal allergy. Characterization of this cellular mechanism could have clinical implications by supporting the development of new diagnoses or treatments for these allergic diseases.

## Materials and Methods

### Ethics

This study was conducted according to the principles expressed in the Declaration of Helsinki. The study was approved by the Institutional Review Board of the University of Tokushima (toku09021).

### Mice

Female C57BL/6J mice (6–8 weeks old) were purchased from CLEA Japan, Inc (Tokyo, Japan). All mice were maintained in specific pathogen-free conditions in our animal facility.

### Histology and Immunohistochemistry

Skin was removed from the mice, fixed with 10% phosphate-buffered formaldehyde (pH 7.2), and prepared for histological examination. Formalin-fixed tissue sections were stained with hematoxylin and eosin. Immunohistochemistry was performed for freshly frozen sections by the biotin-avidin immunoperoxidase method using an avidin-biotin immunoperoxidase complex reagent (Vector Laboratories, Burlingame, CA, USA). Monoclonal antibodies against CD3 and CD19 (eBioscience, San Diego, CA, USA) were used.

### Induction of Ni Allergy

Ni allergy was induced using a modification of a method described previously [24], [25]. To induce a hypersensitivity reaction to Ni, 25 µl of 1 µmol/ml NiCl_2_ with 25 µl of IFA (ICN Biomedicals, Inc., Aurora, OH, USA) was intraperitoneally injected into B6 mice for initial immunization. Two weeks later, mice were administered intradermal injections of Ni, 10 µl of 0.2 µmol/ml NiCl_2_ with CFA (ICN Biomedicals, Inc.) using 28G1/2 needles (TERUMO Tokyo, Japan) for a recall immune response. DTH reactions were determined by measuring the changes in ear thickness at 24 or 48 hours after the challenge [25].

### DC Preparation

DCs were prepared from freshly isolated bone marrow cells as described previously [26]. Briefly, bone marrow cells were seeded in 6-well culture plates (Nunc A/S, Roskilde, Denmark) in RPMI-1640 medium supplemented with 10% heat-inactivated FCS, 2 mM L-glutamine, 100 U/ml penicillin, and 100 µg/ml streptomycin, and then incubated for 1 hour at 37°C in a humidified 5% CO_2_ atmosphere. After nonadherent cells were removed, adherent cells were incubated and changed to 1 ml of fresh medium containing 10 ng/ml GM-CSF (R&D System, Inc., Minneapolis, MN, USA) and 1 ng/ml IL-4 (eBioscience) on days 2, 4, and 6. Dermal DCs were prepared from the skin of female B6 mice. Excised skin was cut into small pieces and the dermal side was exposed to 1.0% trypsin at 37°C for 60 minutes. Epidermis was incubated in 0.025% DNase for 20 minutes at room temperature. After an equal volume of RPMI was added, the solution was swirled for 5 minutes and filtered using a 70 µm cell strainer (BD Biosciences, Franklin Lakes, NJ, USA). The dermal cell suspension that included DCs was washed three times with RPMI, and the resulting pellet was re-suspended. Dermal DCs were enriched by centrifugation using Opti-prep (Invitrogen, Carlsbad, CA, USA).

### Flow Cytometry

Expression levels of surface markers were examined by staining 1×10^6^ cells with 1 µg/ml of antibodies against CD3, CD4, CD8, CD19, CD11c, and MHC class II conjugated with either FITC or PE (eBioscience). Cells were analyzed on FACScan (BD Biosciences).

### Western Blot Analysis

Samples of stimulated or nonstimulated DCs were subjected to sodium dodecyl sulfate-polyacrylamide gel electrophoresis (SDS-PAGE) with 10% acrylamide gel, transferred onto a polyvinylidene fluoride (PVDF) membrane (Bio-Rad Laboratories, Hercules, CA, USA), and the blotted membranes were incubated with anti-MKK6, phospho-MKK6 (Cell Signaling Technology, Inc., Denver, MA, USA), or glyceraldehyde-3-phosphate dehydrogenase (GAPDH) (Santa Cruz Biotechnology, Inc., Santa Cruz, CA, USA) mAbs. Immune complexes were detected using horseradish peroxidase (HRP)-conjugated anti-mouse IgG (Bio-Rad Laboratories) and ECL-plus reagents (Amersham Bioscience Corp. Piscataway, NJ, USA).

### MKK6 Plasmid Construction and Transfection

MKK6 cDNA obtained from RT-PCR was subcloned into an expression vector, pcDNA3.1 (Invitrogen). Transient transfections were carried out using a jet-PEI Mannose reagent (Poly transfection, Illkirch, France) before stimulation. DCs were transfected with the MKK6 plasmid, and then stimulated with either 1 µmol/ml NiCl_2_ or 1 µmol/ml TiO_2_ for 48 hours.

### siRNA for MKK6

MKK6 and control scramble siRNA reagents were obtained from B-bridge International, Inc. (Sunnyvale, CA, USA). Sequences of the oligonucleotides were as follows: MKK6: sense, 5′-CUACAGUAGUGAAGAGAUUTT-3′; antisense, 5′-AAUCUCUUCACUACUGUAGTT-3′ and control: 5′-ATCCGCGCGATAGTACGTA-3′. Using the *in vivo* siRNA transfection kit AteloGene (KOKEN, Tokyo, Japan), siRNA was injected into ear skin according to the manufacturer's instructions [47]. In brief, 10 µM siRNA was mixed with AteloGene and rotated gently at 4°C for 20 minutes. This solution (20 µl) was subcutaneously injected into the ear pinnae. Five days after injection, ear swelling was evaluated.

### Confocal Microscopic Analysis

Skin sheets were isolated from ears of treated mice with 3.8% ammonium thiocyanate, and stained with anti-phospho-MKK6 (Santa Cruz Biotechnology) mAb and MHC class II mAb (eBioscience). These sheets were analyzed using Confocal Laser Microscan (LSM 5 PASCAL; Carl Zeiss. Inc., Oberkochen, Germany).

### Statistical Analysis

Results are given as means ± standard deviations (SD). Comparison was done using Student's *t* test or Mann-Whitney *U* test. Differences were considered statistically significant for *P* values of <0.05.

## Supporting Information

Figure S1 Inflammatory lesions in Ni allergy model. (A) Edema and inflammatory cell infiltrations were observed in the skin tissues of Ni allergy mice. Photos are representative of five mice in each group. Original magnification is ×100 (upper) and ×200 (lower). (B) The number of toluidine blue^+^ cells including mast cell in the lesion of Ni allergy model was significantly increased compared to that of control mice. Toluidine blue staining was performed as described in Methods S1. Photos are representative of three mice in each group. Data are means ± SD of 3 mice. *P<0.05, vs control.
(TIF)

Figure S2 Flow cytometric analysis of immune cells in Ni allergy model. (A) CD4^+^ and CD8^+^ T cells or NK1.1^+^ cells of ear tissues form controls and Ni allergy models were detected by flow cytometry. Results are representative of three mice in each group. (B) NKT cells of spleen, cervical lymph nodes, and ear tissues were detected by using PE-conjugated anti-α∼GalCer mAb-CD1d complex. Results are representative of three mice in each group. (C) CD11c^+^ MHC class II^+^ DCs in cervical lymph nodes (LNs) from control, OVA, TiO_2_, and NiCl_2_-injected mice were analyzed by flow cytometry as described in Methods S1. The results were representative of three to five mice in each group.
(TIF)

Figure S3 Activation of MAPK signaling of DCs by Ni. (A) BMDCs were stimulated with NiCl_2_, LPS or TiO_2_ for 24 hours, phosphorylation of MKK6 and p38, and total MKK6 and p38 protein were detected by Western blot analysis. GAPDH was used as the respective internal control. Results are representative of 3 independent experiments. (B) MKK6 mRNA expression of BMDCs stimulated with NiCl_2_ or LPS was analyzed by real-time PCR as described in Methods S1. Data are shown as relative expressions to β-actin, and are representative of 3 independent experiments.
(TIF)

Figure S4 Cytokine secretions from Ni-stimulated T cells. T cells from cLNs of control, OVA, TiO_2_, and NiCl_2_-injected mice were enriched by negative selection using mAbs (anti-MHC class II, B220, NK1.1, and CD11b) and magnetic beads. The T cells were stimulated with plate-coated anti-CD3 mAb for 24 hours. The secretions of IL-2, IFN-γ, IL-4, and IL-10 in the supernatants were analyzed by ELISA as described in Methods S1. Data are means ± SD of triplicates. *P<0.05, vs control.
(TIF)

Figure S5 Cytokine secretions from Ni-stimulated DCs. BMDCs were stimulated with NiCl_2_, LPS, and TiO_2_ for 24 hours. The cytokine secretions of IL-12, IFN-γ, and IL-10 were detected by ELISA as described in Methods S1. Data are means ± SD of triplicates.
(TIF)

Methods S1 (DOCX)
