# Supplementary figures and images for: A Novel DC Therapy with Manipulation of MKK6 Gene on Nickel Allergy in Mice
Source: PLoS One. 2011 Apr 22;6(4):e19017. doi: 10.1371/journal.pone.0019017 (PMC3081319; doi:10.1371/journal.pone.0019017)

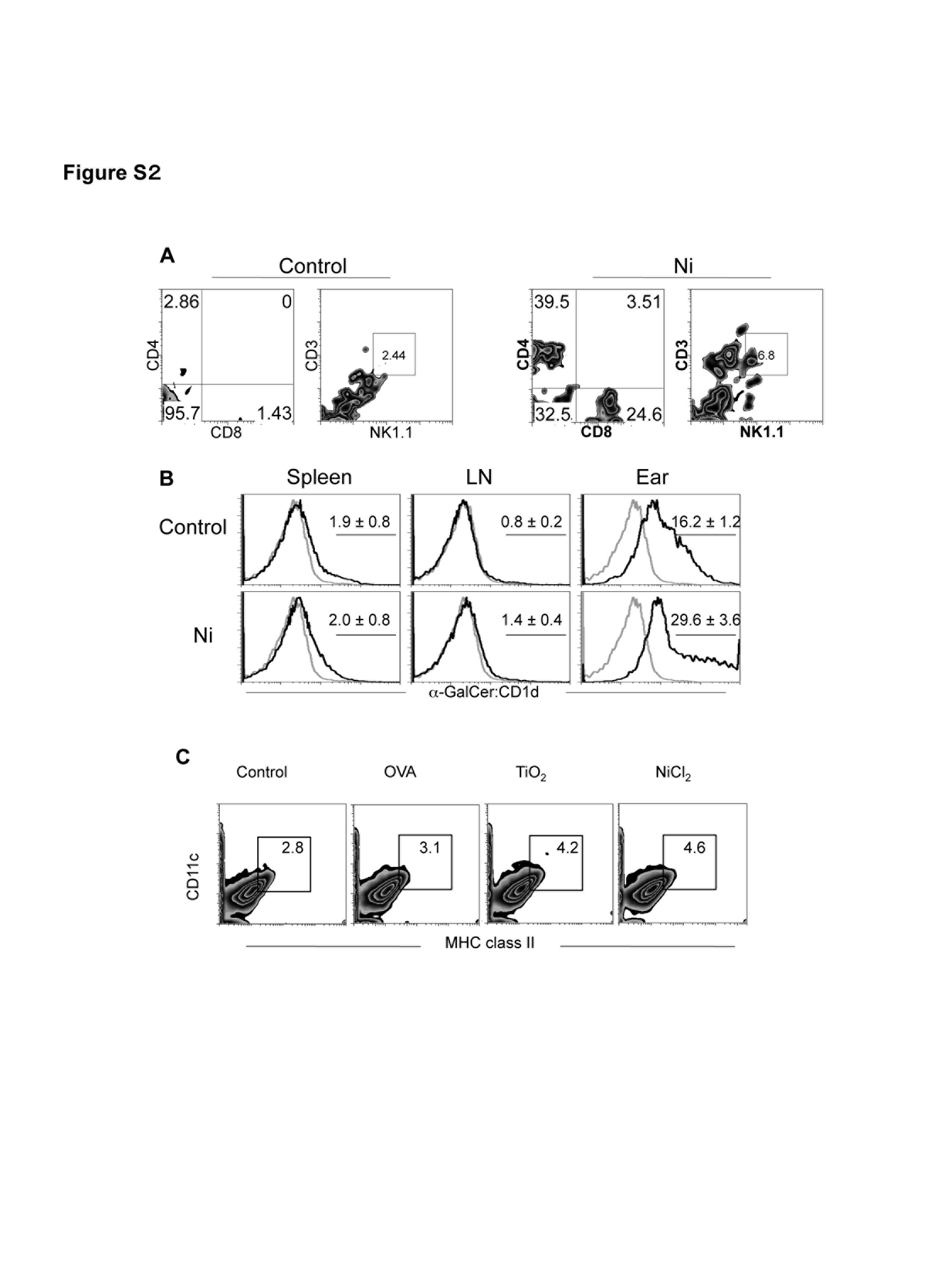

Supplement: Figure S2 — Flow cytometric analysis of immune cells in Ni allergy model. (A) CD4+ and CD8+ T cells or NK1.1+ cells of ear tissues form controls and Ni allergy models were detected by flow cytometry. Results are representative of three mice in each group. (B) NKT cells of spleen, cervical lymph nodes, and ear tissues were detected by using PE-conjugated anti-α∼GalCer mAb-CD1d complex. Results are representative of three mice in each group. (C) CD11c+ MHC class II+ DCs in cervical lymph nodes (LNs) from control, OVA, TiO2, and NiCl2-injected mice were analyzed by flow cytometry as described in Methods S1. The results were representative of three to five mice in each group. (TIF) [file pone.0019017.s002.tif]

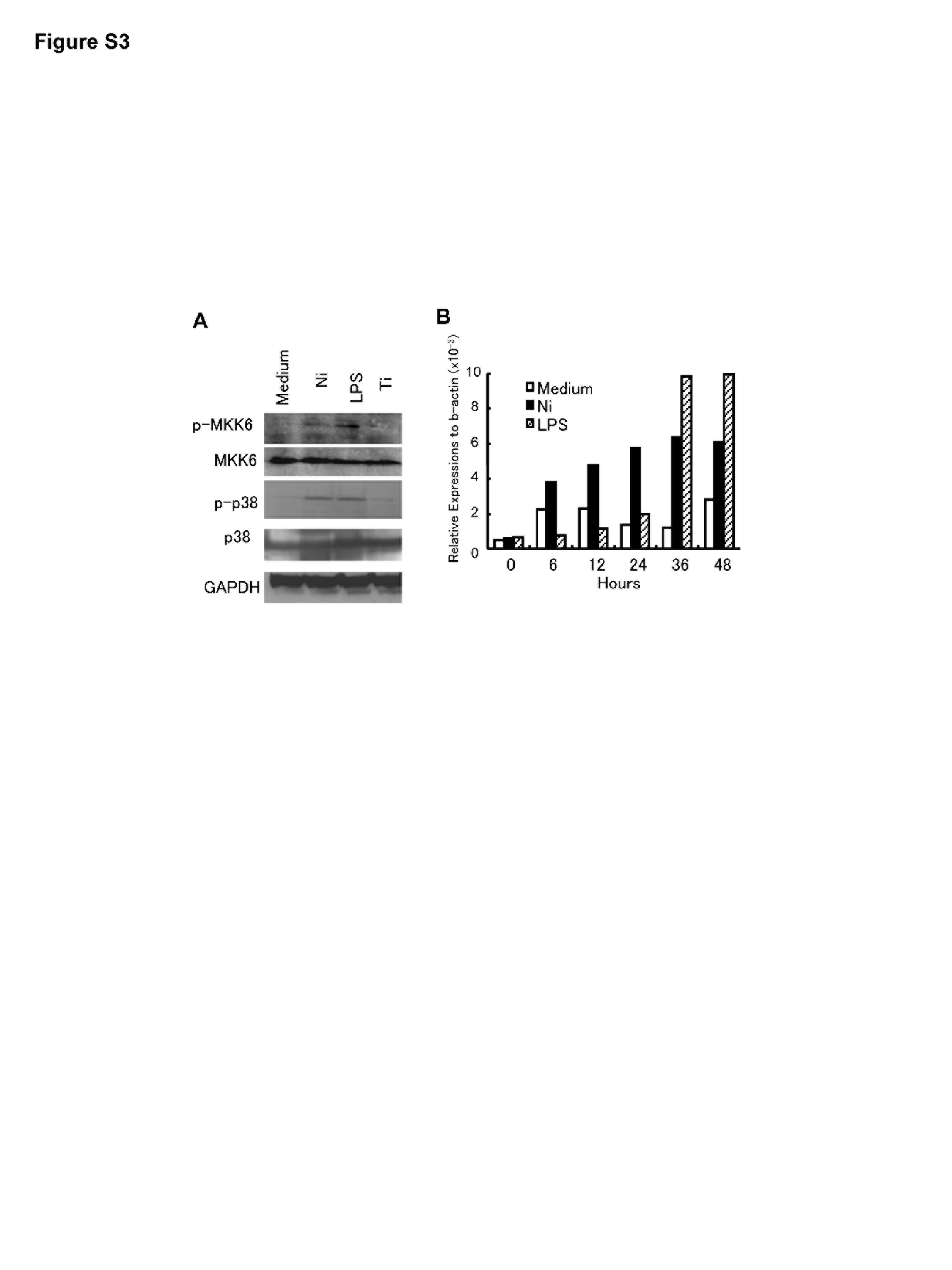

Supplement: Figure S3 — Activation of MAPK signaling of DCs by Ni. (A) BMDCs were stimulated with NiCl2, LPS or TiO2 for 24 hours, phosphorylation of MKK6 and p38, and total MKK6 and p38 protein were detected by Western blot analysis. GAPDH was used as the respective internal control. Results are representative of 3 independent experiments. (B) MKK6 mRNA expression of BMDCs stimulated with NiCl2 or LPS was analyzed by real-time PCR as described in Methods S1. Data are shown as relative expressions to β-actin, and are representative of 3 independent experiments. (TIF) [file pone.0019017.s003.tif]

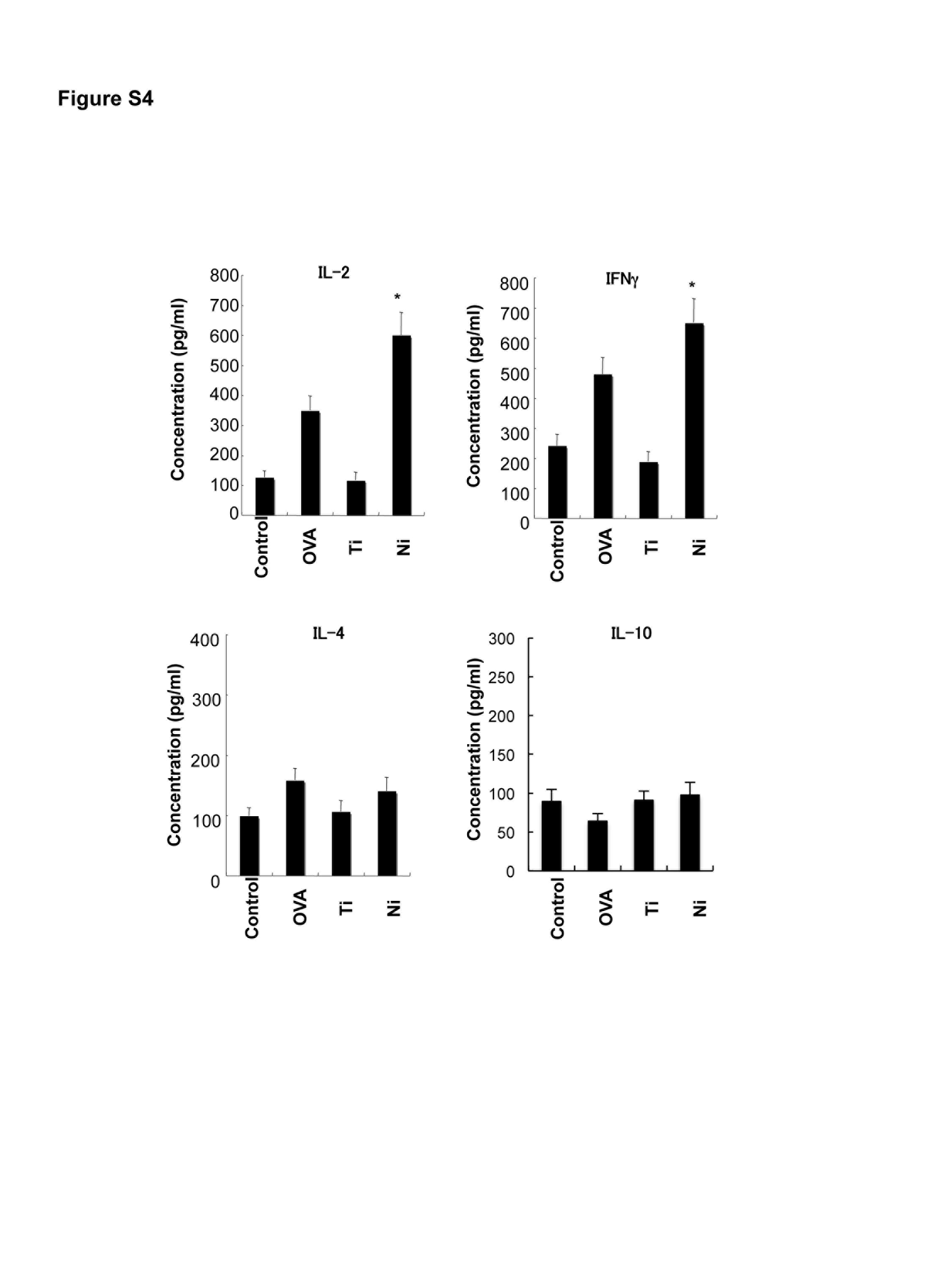

Supplement: Figure S4 — Cytokine secretions from Ni-stimulated T cells. T cells from cLNs of control, OVA, TiO2, and NiCl2-injected mice were enriched by negative selection using mAbs (anti-MHC class II, B220, NK1.1, and CD11b) and magnetic beads. The T cells were stimulated with plate-coated anti-CD3 mAb for 24 hours. The secretions of IL-2, IFN-γ, IL-4, and IL-10 in the supernatants were analyzed by ELISA as described in Methods S1. Data are means ± SD of triplicates. *P<0.05, vs control. (TIF) [file pone.0019017.s004.tif]

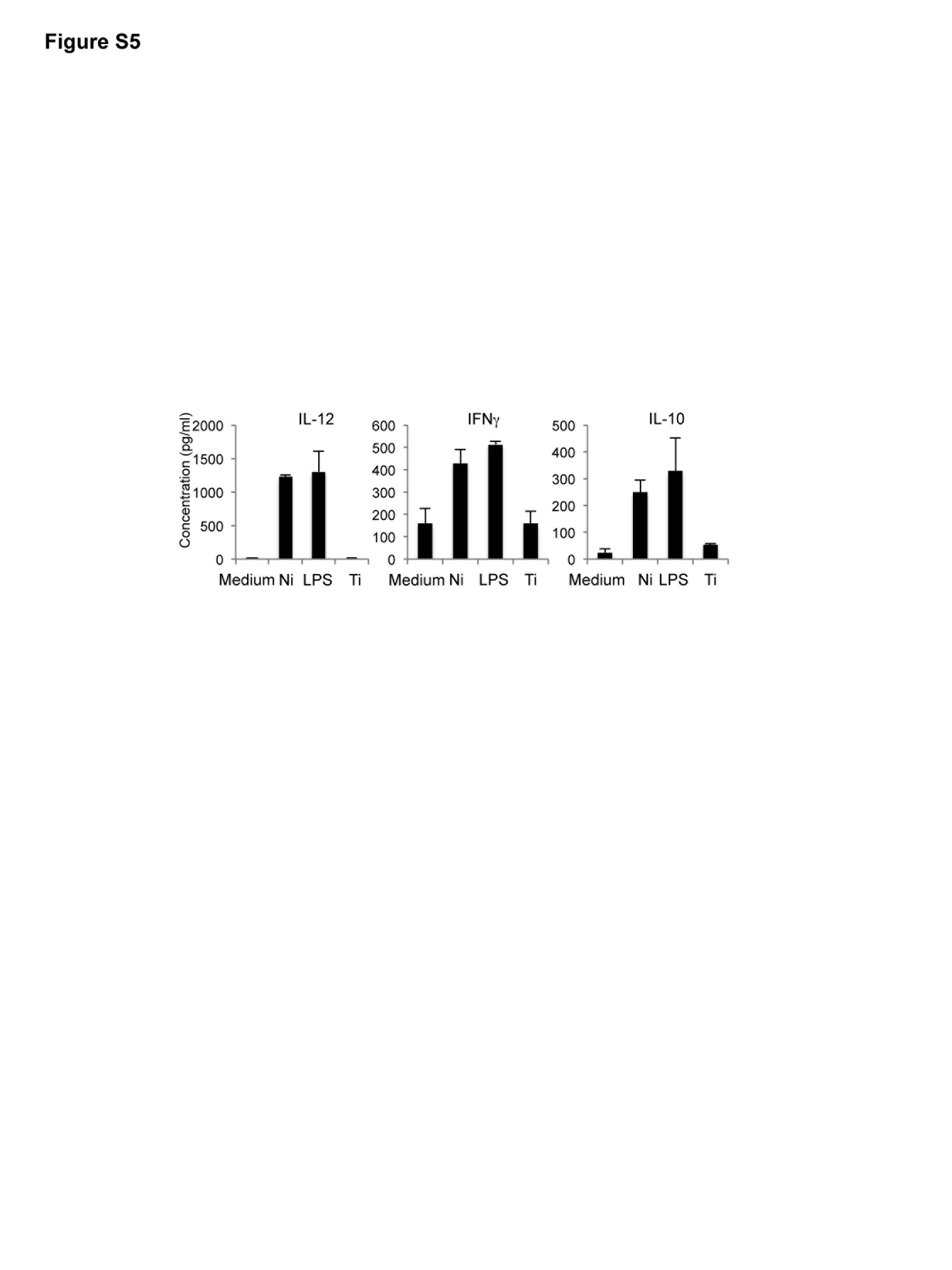

Supplement: Figure S5 — Cytokine secretions from Ni-stimulated DCs. BMDCs were stimulated with NiCl2, LPS, and TiO2 for 24 hours. The cytokine secretions of IL-12, IFN-γ, and IL-10 were detected by ELISA as described in Methods S1. Data are means ± SD of triplicates. (TIF) [file pone.0019017.s005.tif]
