# Supplementary material for: A Novel DC Therapy with Manipulation of MKK6 Gene on Nickel Allergy in Mice
Source: PLoS One. 2011 Apr 22;6(4):e19017. doi: 10.1371/journal.pone.0019017 (PMC3081319; doi:10.1371/journal.pone.0019017)
Supplement: Methods S1 — (DOCX) [file pone.0019017.s006.docx]

**Supplementary Information**

**A novel DC therapy with manipulation of MKK6 gene on nickel allergy in mice**

**Megumi Watanabe^1,2^, Naozumi Ishimaru^1^, Meinar Nur Ashrin^1,2^, Rieko Arakaki^1^, Akiko Yamada^1^, Tetsuo Ichikawa**^2^**, and Yoshio Hayashi**^1^

^1^Department of Oral Molecular Pathology, Institute of Health Biosciences, The University of Tokushima Graduate School, Tokushima, Japan.

^2^Department of Oral Maxillofacial Prosthodontics, Institute of Health Biosciences, The University of Tokushima Graduate School, Tokushima, Japan.

Correspondence

Naozumi Ishimaru, DDS, PhD, Department of Oral Molecular Pathology, Institute of Health Biosciences, The University of Tokushima Graduate School, 3-18-15 Kuramotocho, Tokushima 770-8504, Japan.

**Methods**

**Toluidine blue staining**

Toluidine blue staining was performed using frozen sections of ear tissues.

**Flow cytometric analysis**

Expression levels of surface markers were examined by staining 1×10^6^ cells with 1 μg/ml of antibodies against CD3, CD4, CD8, CD19, NK1.1, CD11c, and α-GalCer-CD1d complex conjugated with either FITC or PE (eBioscience). Cells were analyzed on FACScan (BD Biosciences).

**RNA purification and real-time quantitative RT-PCR**

Total RNA was obtained from DCs with ISOGEN (Wako Pure Chemical, Osaka, Japan) according to the manufacture’s protocol. 5 μg total RNA was used to generate cDNA by Superscript II (GIBCO BRL, Gaithersburg, MD). Transcript levels were measured using PTC-200 DNA Engine Cycler (Bio-Rad Laboratories) with SYBR Premix Ex Taq (Takara). Primers are as follows: MKK6, forward 5’-CCACAGTTAATAGCCAGGAACA-3’ and reverse 5’-ACCCTGTAAACCCACCAATC-3’ ; β-actin, forward 5’-CTCTTTGATGTCACGCACGATTTC-3’ and reverse 5’-GTGGCCGCTCTAGGCACCAA-3’.

**ELISA**

Supernatant samples from stimulated or non-stimulated DCs were tested for IL-12, IFN-γ, and IL-10 productions on ELISA plates using ELISA kit (eBioscience) for each cytokine.

**Western blot Analysis**

The protein extracts of skin tissues were purified using radioimmunoprecipitation assay buffer (50 mM Tris-HCl, pH 7.4, 150 mM NaCl, 1 mM EDTA, 1% NP-40, 1 mM dithiothreitol (DTT), 1 mM phenylmethylsulfonyl fluoride) supplemented with a protease inhibitor cocktail (Sigma Chemical Co., St. Louis, MO), and subjected to SDS-PAGE with 10% acryl-amide gel, transferred to PVDF membrane (Bio-Rad Laboratories, Hercules, CA), and blotted membranes were incubated with each specific antibody, as follows: MKK6 pAb, (Santa Cruz Biotechnology, Santa Cruz, CA), phosphorylated MKK6 (Sigma Chemical, St. Louis, MO) pAb, p38 mAbs (BD PharMingen, San Diego, CA), and phosphorylated p38 mAb, (Sigma Chemical, St. Louis, MO). Immune complexes were detected with HRP–conjugated anti-mouse IgG or anti-rabbit IgG (Bio-Rad Laboratories) and ECL-plus regents (Amersham Bioscience Corp. Piscataway, NJ).
